# Guided-Mode-Leaky-Mode-Guided-Mode Fiber Interferometer and Its High Sensitivity Refractive Index Sensing Technology

**DOI:** 10.3390/s16060801

**Published:** 2016-06-01

**Authors:** Qi Wang, Chunyue Li, Chengwu Zhao, Weizheng Li

**Affiliations:** 1 College of Information Science and Engineering, Northeastern University, Shenyang 110819, China; 20123553@stu.neu.edu.cn (C.L.); 20123645@stu.neu.edu.cn (C.Z.); 20133541@stu.neu.edu.cn (W.L.); 2 State Key Laboratory of Synthetical Automation for Process Industries, Northeastern University, Shenyang 110819, China

**Keywords:** optical fiber sensor, refractive index measurement, Micro-Nano fiber, Mach-Zehnder interferometer, high sensitivity

## Abstract

A cascaded symmetrical dual-taper Mach-Zehnder interferometer structure based on guided-mode and leaky-mode interference is proposed in this paper. Firstly, the interference spectrum characteristics of interferometer has been analyzed by the Finite Difference-Beam Propagation Method (FD-BPM). When the diameter of taper waist is 20 μm–30 μm, dual-taper length is 1 mm and taper distance is 4 cm–6 cm, the spectral contrast is higher, which is suitable for sensing. Secondly, experimental research on refractive index sensitivity is carried out. A refractive index sensitivity of 62.78 nm/RIU (refractive index unit) can achieved in the RI range of 1.3333–1.3792 (0%~25% NaCl solution), when the sensor structure parameters meet the following conditions: diameter of taper waist is 24 μm, dual-taper length is 837 μm and taper distance is 5.5 cm. The spectrum contrast is 0.8 and measurement resolution is 1.6 × 10^−5^ RIU. The simulation analysis is highly consistent with experimental results. Research shows that the sensor has promising application in low RI fields where high-precision measurement is required due to its high sensitivity and stability.

## 1. Introduction

The refractive index (RI) is a significant physical parameter to reflect the characteristics of material [1,2]. RI measurement is also important in many application fields such as the detection of solution concentration in the chemical field, water quality monitoring in the environmental field, and real-time monitoring cell culture medium in the biology field. In recent years, because of the advantages of compact structure, small size, light weight, rapid response, high sensitivity, low loss, anti-electromagnetic interference and applicability for remote sensing, fiber optics sensing technology have been widely studied and are becoming a hot research topic [2,3,4]. There have been many reports on optical fiber sensors which were used for measuring liquid RI, but the sensitivities of these sensors are not high enough in low RI (near 1.33) liquid or the structures are complex [5]. In 2008, Zhaobing Tian put forward a new type fiber RI sensors based on two positive-cascaded 3 dB tapers Mach-Zehnder interferometer by optical fiber fusion splicer [6]. The experiment shows that the spectrum contrast is 23.4 dB and RI sensitivity is 17.1 nm/RIU when L is 36 mm. In 2009, Ping Lu made two conical structures in a single mode fiber to build a Mach-Zehnder interferometer [7] for simultaneous measurement of RI and temperature, and the RI sensitivity can reach 26.087 nm/RIU. In 2011, Rao introduced a piece of slow-taper and weak-pulling taper area into the middle of two cascaded tapers to form a "sandwich" structure [8]. The principle is that the middle structure can enhance the evanescent field of optical energy and increase the exposed energy of the light field for achieving the improvement of RI sensing sensitivity, and a RI sensitivity of 28.6 nm/RIU has been realized in the RI range of 1.3320–1.3890.

Although these sensors show high sensitivities in the high RI measurements field (near the RI of silica), it can hardly meet the high-precision measurement requirement in low RI measurement field. In this paper, we proposed a Mach-Zehnder interferometer structure based on the interference between modes. When the diameter of the taper waist is 24 μm, dual-taper length is 837 μm and taper distance is 5.5 cm, the RI sensitivity is 62.78 nm/RIU in the RI range of 1.3333–1.3792. The spectrum contrast is 0.8 and measurement resolution is 3 × 10^−4^ RIU. The results show that simulation results and experimental are highly consistent. In this paper, the fabrication process of the sensor is simple and fast, the cost is low, and the RI sensitivity of the sensor is higher, which is expected to be widely used in the field of stress [9,10], temperature [11], and so on.

## 2. Preparation and Principle

### 2.1. Preparation of Fiber Interferometer

**Figure 1:**
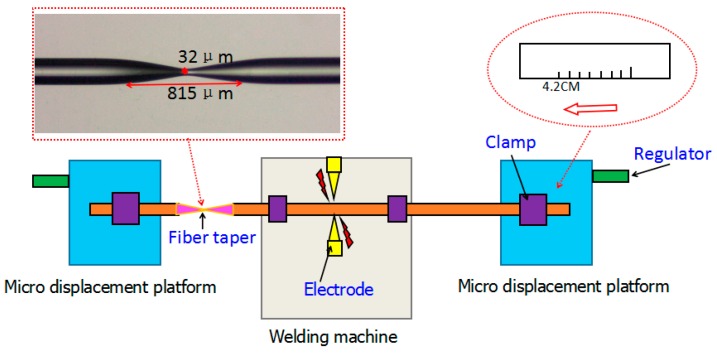
Cascaded symmetrical dual-taper preparation of Mach-Zehnder interferometer.

A simple and feasible, low cost and good repeatability fiber dual-taper making process should be devised as soon as possible [12]. A new method is proposed in this paper, which only needs a fusion splicer (S178, FITEL, FURUKAWA ELECTRIC CO., LTD., Tokyo, Japan) and a high precision micro displacement platform (NFP-x462, ZOLIX, ZOLIXINSTRUMENTS CO.LTD., Beijing, China). The fabrication process of the interferometer is shown in Figure 1.

Firstly, a 5 cm length coating layer of single-mode fiber was removed by using a miller clamp. Then, we cleaned the surface of the fiber by using lens paper with alcohol and pretreated the fiber without coating layer in high temperature for 5 min in a fusion splicer. Finally, we used the fusion splicer to control tapered fiber structure parameters such as taper-area length, taper angle and diameter of taper waist. In the first experiment, we got a taper with taper area length of 816 μm and taper waist diameter of 32 μm, which is shown in Figure 1. The transmitted power is −26.03 dBm when the fiber was integrated, and the total transmitted power is −29.39 dBm after drawing a dual-taper structure. The interference phenomenon appeared when we made a second taper which is 4.2 cm away from the first one, and the total power of the interferometer is −32.23 dBm. The spectrum of backward incidence is basically consistent with that of forward incidence, which proved that the performance of the interferometer structure above is stable.

### 2.2. Sensing Principle of Fiber Interferometer

**Figure 2:**
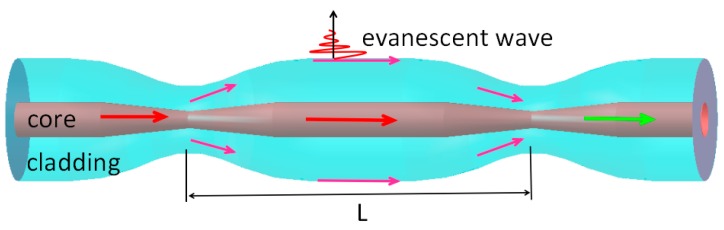
Principle diagram of dual-taper Mach-Zehnder interferometer.

In this paper, the cascaded dual-taper fiber structure forms a Mach-Zehnder interferometer. The fundamental mode in fiber core excites high-order modes in the first taper area and couples into the cladding. Then the high-order modes transmitted in cladding produce a strong evanescent field in the interface of cladding and external environment. Fundamental mode and high-order modes with external environment RI information will couple with each other. Due to the different effective RI between cladding modes and core mode, a phase difference will come up after the light transmitting a distance L. So this cascaded dual-taper structure forms a Mach-Zehnder interferometer. Figure 2 shows the tapered fiber Mach-Zehnder interferometer, and the interval length of two tapers (interference length) is L.

Because many transport modes exist in the cladding and only the fundamental mode exists in the fiber core, usually one of the cladding modes interferences with fundamental mode when analyzing the interference pattern. Therefore, the interference intensity between fundamental mode and cladding mode can be expressed with double-beam interference principle [13,14].

(1) $$I=I_{1}+I_{2}+2\sqrt{I_{1}I_{2}}\cos(\varphi +\varphi_{0})$$

(2) $$\varphi =\frac{2\pi (n_{\text{co}}-n_{\text{cl}})L}{\lambda }=\frac{2\pi \Delta n_{\text{eff}}L}{\lambda }$$

Among them, $I_{1}$ and $I_{2}$ are beam intensity of fundamental mode and cladding mode, φ is the phase difference between fundamental mode and cladding mode, $\varphi_{0}$ is the initial phase, $n_{\text{co}}$ is the RI of fiber core mode and $n_{\text{cl}}$ is the RI of cladding mode, $\Delta n_{\text{eff}}$ is the difference of RIs, L is the length of interferometer, λ is the input wavelength. From Equation (1), we can see that the contrast ratio of interference fringe is depended on the ratio between $I_{1}$ and $I_{2}$.

The contrast ratio of interference fringe can reach maximum value when the phase difference satisfies the condition of $\varphi +\varphi_{0}=(2m+1)\pi$, and the light intensity of interference reach minimum value. The corresponding interference dip wavelength can be expressed as Equation (3).

(3) $$\lambda_{m}=\frac{2\Delta n_{\text{eff}}L}{2m+1}$$

The RI of the solution around the fiber will increase when the sensor is immersed in a solution, it will cause the change of cladding mode normalized frequency. Then the mode field distribution and effective RI will change. Because the fundamental mode of the fiber core is almost not affected by the RI change of external environment, the effective RI difference between fiber core and cladding mode will change, which will cause a shift of interference spectrum dip. Thus, we can realize an accurate measurement for external environment RI. The RI measurement sensitivity is associated with Mach-Zehnder interferometer structure. Moreover, the measuring range of Mach-Zehnder interferometer is also an important indicator which is usually measured by the distance between adjacent dips. The greater the distance between dips, the greater the spectrum measurement range will be, and the more difficult it will be to generate mixing. In theory, the free spectrum range $\Delta \lambda_{m}$ can be expressed as Equation (4).

(4) $$\Delta \lambda_{m}=\lambda_{m}-\lambda_{m-1}\approx \frac{\lambda^{2}}{\Delta n_{\text{eff}}\cdot L}$$

## 3. Simulation Analysis of Fiber Mach-Zehnder Interferometer

### 3.1. Spectrum Characteristic Simulation Analysis of Fiber Mach-Zehnder Interferometer

**Figure 3:**
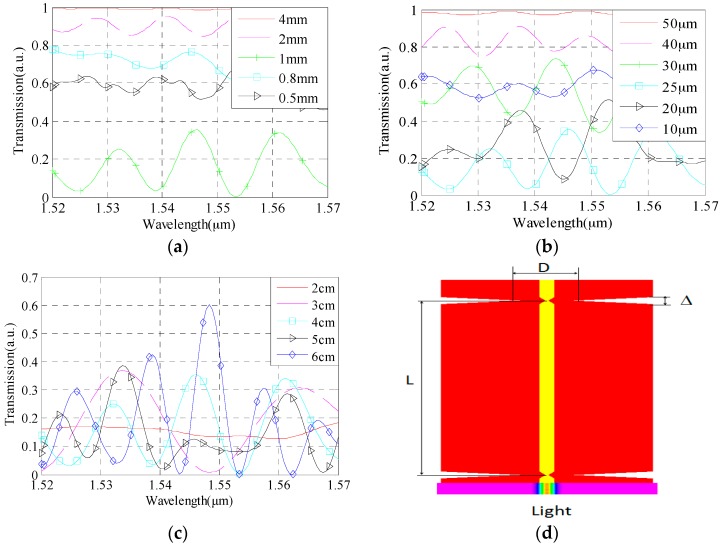
The simulation model and interference spectrum with different structural parameters. (**a**) Different taper area length ∆; (**b**) Different taper waist diameter D; (**c**) Different cone distance L corresponding; (**d**) The simulation model.

We analyzed the cascaded dual-taper interference spectrum and RI sensitivity. The adjustable parameters of the structure mainly include cascaded dual-taper area length ∆, diameter of taper waist D and distance between two tapers L, as shown in Figure 3d.

Figure 3a shows the influence of taper length on spectrum contrast. Firstly, we simulate and analyze the distribution of field excited by tapers, and find that the excitation is better when the diameter of taper is 25 μm. So, we set the diameter as 25 μm, and the distance between two tapers is 4 cm. We get the interference spectrum of cascaded dual-taper when the taper length varies among 0.5 mm, 0.8 mm, 1 mm, 2 mm, and 4 mm. As shown in Figure 3a, the transmitted spectrum does not show visible interference when the length of taper is 4 mm. When the taper length decreases to 2 mm, visible interference appears. However, the contrast of the spectrum is low, and we can not find the shift of taper length and dip with the change of RI. Moreover, all of the change cannot be found when the taper length decreases to 500 μm. Only when the taper length is 1 mm, are the contrast of interference spectrum and free spectrum range better for the study of the change of taper length and dip with RI variation. Because of these, we mainly study RI sensing characteristics of cascaded dual-taper whose length is 1 mm.

Figure 3b shows taper diameter influence on spectrum. According to the analysis above, we set the taper length as 1 mm, the distance between two tapers as 4 cm. From Figure 3b we can find that when the taper diameter is 50 μm, the spectrum contrast is too low. When the taper diameter decreases gradually, the spectrum contrast becomes better. In this process, we will get an ideal contrast when the taper diameter varies from 30 μm to 20 μm. So we mainly research RI sensing characteristics with the taper diameter varying from 30 μm to 20 μm.

Figure 3c shows the distance of two tapers influence on spectrum contrast. As in the simulation described above, it will get a better proportion of light coupling when the diameter is 25 μm. In this paper, we set the length of the taper as 1 mm, diameter of the taper as 25 μm. We can obtain interference spectrum when L varies among 4 cm, 3 cm, 5 cm, 6 cm and 2 cm. From Figure 3c we can see that the taper distance gradually increases from 2 cm to 6 cm, the interference spectrum is becoming more and more intensive, and the free spectrum range is becoming smaller and smaller, which is consistent with the theoretical analysis results. Although the RI measurement sensitivity of cascade dual-taper structure will increase with the increase of tapers distance, the taper distance can not be too long, because sensor mechanical strength will decrease as the taper distance increases.

According to the simulation analysis, we obtained the better cascade dual-taper structure parameters, and taper waist diameter range is determined as 20 μm–30 μm and taper distance range is determined as 4 cm–6 cm, then we analyzed the cascade dual-taper interferometer RI sensitivity with different structural parameters.

### 3.2. Simulation Analysis on Mach-Zehnder Sensor Refractive Index Sensitivity

In this experiment, NaCl solution is more easy to achieve the mass percentage concentration range 0%–25%, the corresponding RI range is 1.3333–1.3792. In this paper, we set the solution RI of 1.34, 1.35, 1.36, 1.37, 1.38 respectively. The length of the taper area is 1 mm, diameter of the taper is 30 μm, 25 μm and 20 μm respectively, and the distance of the tapers is 5 cm.

**Figure 4:**
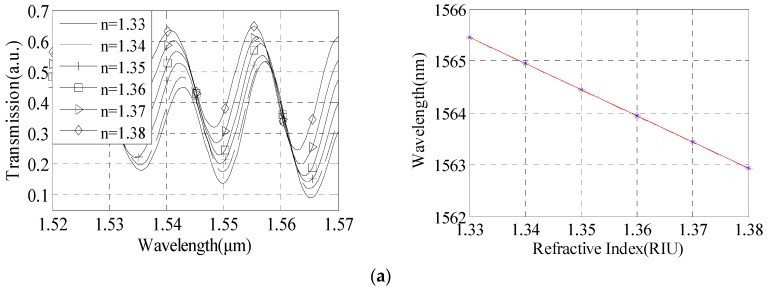

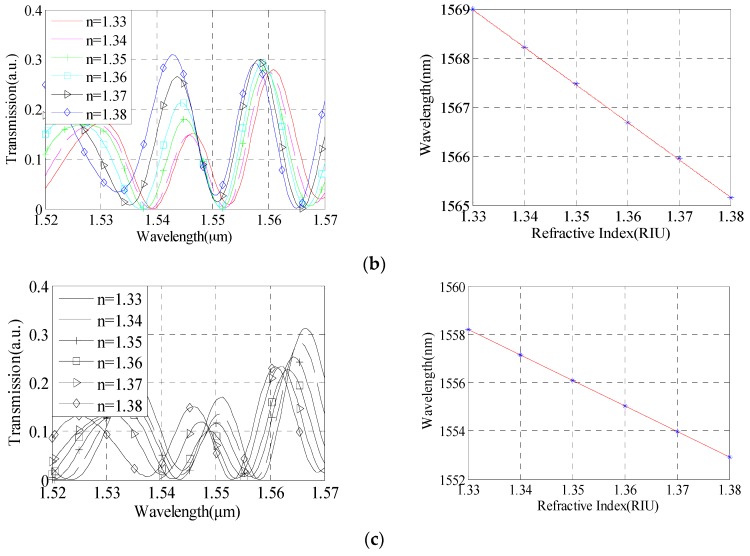
Refractive index measurement sensitivity analysis with different taper waist diameters. (**a**) D = 30 μm; (**b**) D = 25 μm; (**c**) D = 20 μm.

We obtained the transmission spectrum of different RIs and analyzed the sensitivities of the sensor with different structure parameters. The linear fitting relationships between wavelength of interference valley and RI are shown in Figure 4a–c, and the corresponding RI measurement sensitivities can reach 50.5 nm/RIU, 76.3 nm/RIU, and 105.3 nm/RIU, respectively. Thus, in the diameter range of 20 μm to 30 μm, the smaller taper diameter, the higher sensitivity.

Therefore, we needed to fabricate a series of cascade dual-taper structures. Figure 4b is the interference spectrum when taper waist diameter is 25 μm. There are only three dips in the wavelength range of 1520 nm–1570 nm, which proves that the structure parameters of free spectrum range is better.

**Figure 5:**
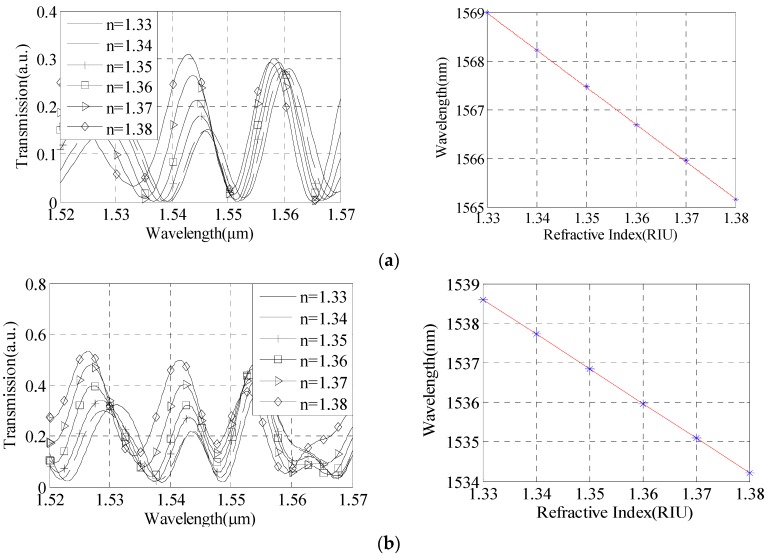
Refractive index measurement sensitivity analysis with different distance of the tapers. (**a**) L = 4 cm; (**b**) L = 6 cm.

The length of the taper area is 1 mm, the distance of the tapers is 4 cm, 5 cm and 6 cm respectively, and the diameter of the taper is 25 μm. We obtained the transmission spectrum of different RIs and analyzed the sensitivities of the sensor with different structure parameters. The linear fitting relationships between wavelength of interference valley and RI are shown in Figure 4b and Figure 5a,b, and the corresponding RI measurement sensitivities can reach 46.1 nm/RIU, 76.3 nm/RIU, and 87.5 nm/RIU, respectively. Thus, in the tapers distance of 4 cm to 6 cm, the longer taper distance has the highest sensitivity.

## 4. Experiment Results Analysis on Refractive Index Measurement

**Figure 6:**
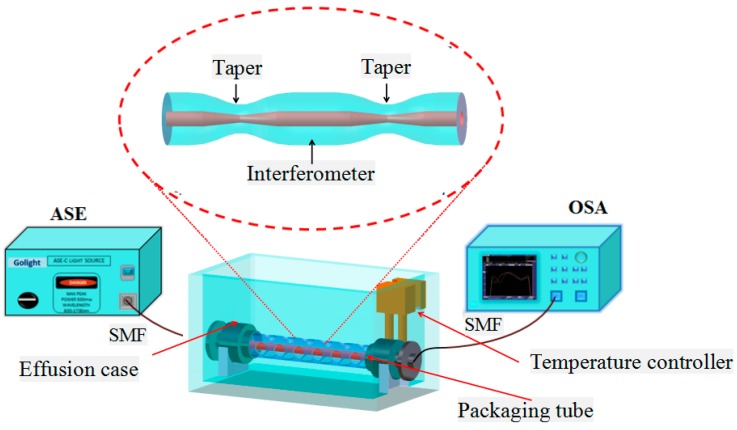
Laboratory equipment schematic of refractive index sensing system.

The experimental device based on cascaded dual-taper Mach-Zehnder interferometer is shown in Figure 6. The experimental system includes an ASE broadband light source with a wavelength range of 1520 nm–1570 nm, an optical spectrum analyzer (AQ6370B, YOKOGAWA, Yokogawa Electric Corporation, Tokyo, Japan) and a liquid box with temperature controller which ensures that the experiment is carried out under constant temperature.

The NaCl solution was used as a detected solution, and the corresponding relationship between RI and solution concentration was obtained as shown in Table 1 [15].

Based on the theory and simulation analysis, a series of experiments were carried out to study the relationship between RI sensitivity and structure parameters of the sensor. When the diameter of taper waist is less than 20 μm, the fiber is broken easily, and encapsulation process is complex. Therefore, we make an interferometer with a diameter of 20 μm–30 μm in our experiments. The interferometer with a diameter of 25 μm had different taper distances of 4 cm–6 cm. The results show that the experimental results are consistent with the simulation.

**Figure 7:**
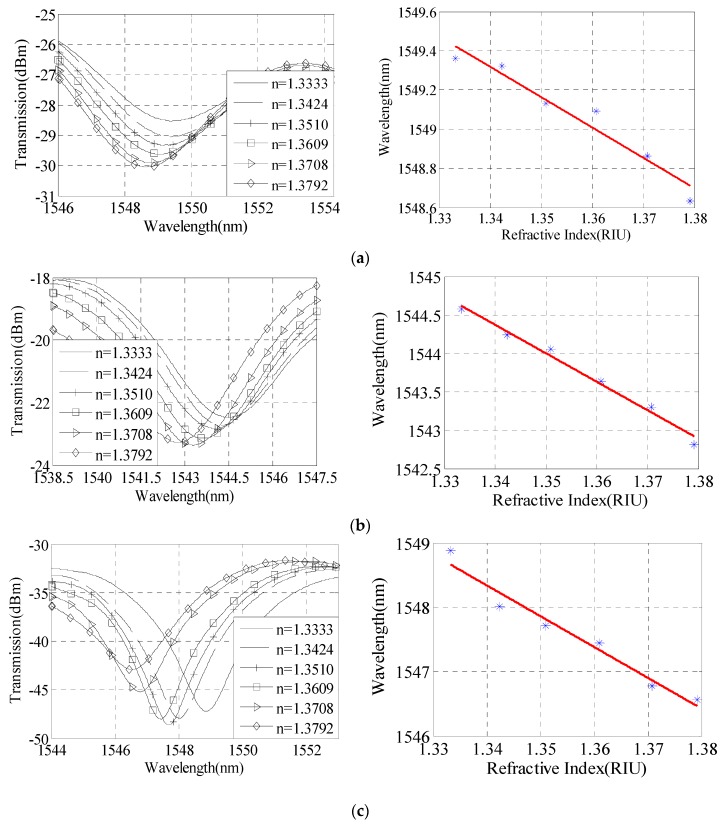
Refractive index measurement sensitivity analysis with different taper waist diameters. (**a**) D = 30 μm; (**b**) D = 25 μm; (**c**) D = 20 μm.

**Figure 8:**
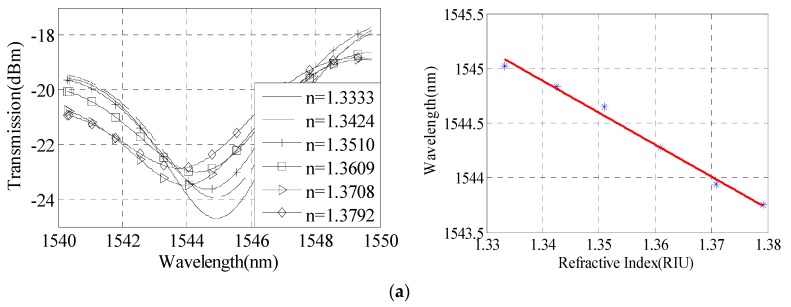

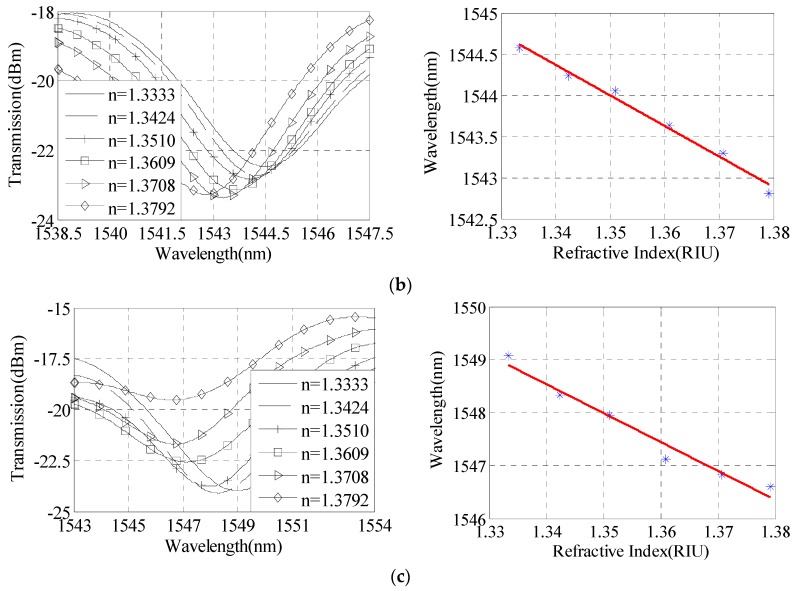
Refractive index measurement sensitivity analysis with different tapers distances. (**a**) L = 4 cm; (**b**) L = 5 cm; (**c**) L = 6 cm.

Figure 7 is the sensor sensitivity with taper waist diameter of 20 μm–30 μm, taper distance of 5 cm, and taper area length of 818 μm. When taper waist diameter D is 30 μm, 25 μm, and 20 μm, the corresponding RI sensitivity is 47.3 nm/RIU, 67.1 nm/RIU, and 96.5 nm/RIU, respectively. The RI measuring sensitivity reduces linearly with the increase of D. Figure 8 is the sensor sensitivity with taper waist diameter of 25 μm, taper distance of 4.0 cm-6.0 cm, taper area length of 818 μm. When taper distance L is 4 cm, 5 cm and 6 cm, the corresponding RI sensitivity is 44.3 nm/RIU, 67.1 nm/RIU, and 81.5 nm/RIU, respectively. Considering the encapsulation, mechanical strength, transmission loss, L is bigger and more conducive to sensing.

In order to reach the higher refractive index sensitivity, we reduced the taper waist diameter as far as possible. The experimental results show that a stable taper waist diameter of 24 μm can be obtained by the fusion splicer. Through the continuous experiments, we finally made a cascade dual-taper interferometer with a taper waist diameter of 24 μm, taper distance of 5.5 cm, and taper area length of 837 μm. The sensitivity and spectrum characteristics of the RI sensor are shown in Figure 8.

**Figure 9:**
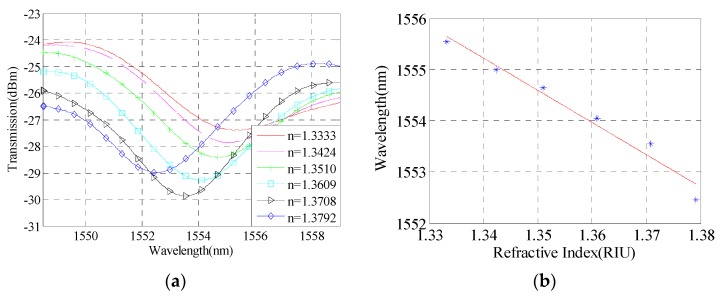
Sensitivity analysis with taper waist diameter of 24 μm, taper distance of 5.5 cm, and taper area length of 837 μm. (**a**) Interference spectrum; (**b**) Sensitivity of refractive index measurement.

As shown in Figure 9a, the interference spectrum shows a red shift with the increasing of solution RI, and when the RI increases from 1.3333 to 1.3792, the wavelength of interference dip moves from 1552.455 nm to 1555.545 nm. The linear relationship is: y=−62.78x+1636. The relationship between interference dip and the RI is obtained by Figure 9b. The RI measurement sensitivity of the sensor can reach 62.78 nm/RIU.

## 5. Conclusions

In this paper, we proposed a cascaded symmetrical dual-taper Mach-Zehnder interferometer based on interference between the guided-mode and leaky-mode mode. A simple and accurate preparation method of fiber taper was designed, and the influence of taper length, taper distance, and taper waist diameter on the spectrum characteristics and RI measurement sensitivities were simulated. In the experiment, the diameter of the taper is 24 μm, length of taper area is 837 μm and taper distance is 5.5 cm. The experimental results show that the RI measurement sensitivity can reach 62.78 nm/RIU in the range of 1.3333~1.3792. The resolution can reach 1.6 × 10^−5^ RIU when using an OSA with a resolution of 1 pm. Due to the advantages of simple fabrication, low cost, and high sensitivity, the sensor promises to have wide applications in the field of stress, temperature, and so on.

## Figures and Tables

**Table 1:** NaCl Solution refractive index table along with the change of concentration.

*c*/%	0	5	10	15	20	25
Refractive Index	1.3333	1.3424	1.3510	1.3609	1.3708	1.3792
